# A Low-Cost Serratus Anterior Plane Block Phantom With a Thoracodorsal Artery Analogue and Pleural-Breach Feedback

**DOI:** 10.7759/cureus.119289

**Published:** 2026-11-07

**Authors:** Chinnu Prince, Samama Rafi, Himanshu G Mirani

**Affiliations:** 1 Emergency Department, Midland Metropolitan University Hospital, Birmingham, GBR

**Keywords:** emergency medicine, phantom, point-of-care ultrasound, regional anaesthesia, rib fractures, serratus anterior plane block, simulation

## Abstract

Ultrasound-guided serratus anterior plane block (SAPB) is an evidence-supported component of multimodal analgesia for rib fractures, but training requires learners to integrate chest-wall sonoanatomy with safe needle control near vascular and pleural structures. We describe a low-cost part-task trainer, meaning a simulator designed to rehearse selected components of a procedure rather than the entire clinical encounter, and propose a staged pathway from simulation to supervised patient practice. Four wooden stacking blocks support a tongue-depressor scaffold. Transverse tongue depressors represent ribs, and a water-filled examination glove sits immediately beneath them as a simulated pleural safety boundary. Two chicken fillets represent serratus anterior and latissimus dorsi, with a single layer of cling film creating the superficial fascial plane. A fluid-filled thermometer probe cover placed in this plane represents the thoracodorsal artery. The primary task is superficial SAPB, with in-plane needle visualisation, vessel avoidance and hydrodissection. An optional deep-plane station is designed to provide immediate visible feedback if an unsafe trajectory breaches the water-filled glove; it does not simulate pneumothorax physiology. One final prototype was evaluated in a proof-of-concept bench session. Post hoc estimates from archived ultrasound images placed the superficial target plane at approximately 8-10 mm, and the rib surrogates at approximately 18-22 mm depth. No validity, reliability, durability or learner-outcome testing was performed. The model and proposed seven-step pathway are therefore offered as a starting point for structured preclinical preparation, not as a stand-alone competency or entrustment tool.

## Introduction

Rib-fracture pain can impair ventilation, coughing and mobilisation. In the SABRE randomised clinical trial, adding an ultrasound-guided serratus anterior plane block (SAPB) to protocolised rib-fracture care increased the proportion of patients achieving an early meaningful reduction in pain and reduced in-hospital opioid use [1]. SAPB is, therefore, best considered an evidence-supported component of multimodal chest-wall analgesia rather than a universal replacement for other analgesic strategies. 

SAPB is a fascial plane block, in which local anaesthetic is deposited within a tissue plane to bathe nerves traversing that plane rather than targeting a single named nerve. The block is commonly performed at approximately the fifth rib in the midaxillary region, with the patient supine or lateral and the ipsilateral arm abducted to expose the lateral chest wall. In the original volunteer description, the fifth rib was identified at the midaxillary line with a high-frequency linear transducer, and the target plane lay approximately 1-2 cm below the skin. Sensory change extended broadly across the anterolateral hemithorax, reported approximately from T2 to T9 [2]. 

Two approaches are described. In the superficial approach, local anaesthetic is deposited between latissimus dorsi and serratus anterior; in the deep approach, it is deposited between serratus anterior and the ribs [2]. The thoracodorsal artery, usually a terminal branch of the subscapular artery, courses in the plane between latissimus dorsi and serratus anterior and is a useful sonographic landmark for the superficial approach [2,3]. Computed tomography angiography data report a mean thoracodorsal artery origin diameter of approximately 2-3 mm [3]. Inadvertent vascular puncture may cause bleeding or haematoma and creates the possibility of intravascular local anaesthetic injection; therefore, vessel identification and needle-path planning are relevant safety behaviours, alongside standard measures to prevent and recognise local anaesthetic systemic toxicity [4]. 

Pleural injury after SAPB appears uncommon and its true incidence is not well quantified. A 2020 systematic review identified no block-related complications among 875 blocks performed in 785 patients, including no reported pneumothorax; a subsequently published case report nevertheless documents pneumothorax after a deep SAPB [5,6]. The rationale for emphasising pleural avoidance in training is, therefore, not that pneumothorax is common, but that the pleura is a nearby safety-critical structure and an avoidable deep needle trajectory can have serious consequences. 

The second challenge is educational. Ultrasound-guided regional anaesthesia requires simultaneous sonoanatomy recognition, probe control, hand-eye coordination and needle-tip tracking. In a randomised study of novice anaesthesia residents, one hour of low-fidelity simulation training improved early block-room performance; eight of 10 simulation-trained residents versus four of 10 conventionally trained residents achieved proficiency during a three-week rotation using cumulative-sum analysis against a predetermined 30% acceptable failure rate [7]. SAPB-specific mastery learning has also been demonstrated in emergency medicine residents using a 19-item procedural checklist and a predefined minimum passing standard [8]. These findings support simulation as preparation for supervised practice, but not as a replacement for clinical assessment. 

The intended learners for the present pathway are UK emergency medicine trainees working within local point-of-care ultrasound and regional anaesthesia governance. The principles are transferable to anaesthesia trainees and other health systems if local credentialing requirements are substituted. We use the term in-plane needling to mean advancing the needle within the ultrasound imaging plane so that the shaft and tip can be followed, hydrodissection to mean injection of fluid to separate adjacent tissue layers and confirm the target plane, and entrustment to mean a supervisor's decision that a learner may perform a task at a defined level of supervision. The Royal College of Emergency Medicine (RCEM) point-of-care ultrasound framework provides a relevant UK precedent for integrating simulation, image acquisition and workplace evidence when judging competence [9]. 

Low-cost SAPB trainers already exist. Odom et al. described a chicken-breast and pork-rib model specifically for SAPB [10], while Naraghi et al. used pork, meat glue and fluid-filled straws to reproduce several regional anaesthesia targets, including the serratus plane [11]. Micheller et al. described a broader low-fidelity meat-based ultrasound-guided nerve block model [12], and more recent work has demonstrated reusable ballistic-gel phantoms for SAPB and other blocks [13]. Our aim was not to claim the first low-cost SAPB phantom, but to adapt these principles to a readily assembled emergency department model that adds a named thoracodorsal artery analogue, a deliberately vulnerable pleural safety boundary and a proposed seven-step pathway in which phantom performance gates progression to directly supervised patient procedures. 

## Technical report

Design rationale and iteration 

The design evolved from an initial consideration of foam, felt and cardboard components. Foam and felt were not used in the final ultrasound path because trapped air and material interfaces were expected to degrade acoustic transmission. Cardboard was avoided as the principal scaffold because the model deliberately incorporates water beneath the tissue stack and requires a rigid platform that tolerates wet handling. Wooden stacking-game blocks were, therefore, used as four legs, with tongue depressors taped across them to form the frame. Tongue depressors were also selected instead of animal ribs to reduce handling and disposal of bone and to provide a simple, firm depth stop. A fluid-filled thermometer probe cover was chosen as the vascular analogue because it is narrow, readily available in clinical environments and can span the ultrasound window as a discrete tubular target. These were pragmatic design choices rather than formal head-to-head material tests. 

Model construction and materials 

One final prototype was constructed inside a waterproof vomit bowl. The build used the components shown in Figure 1 and Table 1. Because exact scaffold dimensions, glove fill volume and assembly time were not prospectively recorded during this proof-of-concept build, these values are not reconstructed retrospectively. Table 1, therefore, separates components that were directly used from recommended replication targets intended to standardise future builds.

**Figure 1:**
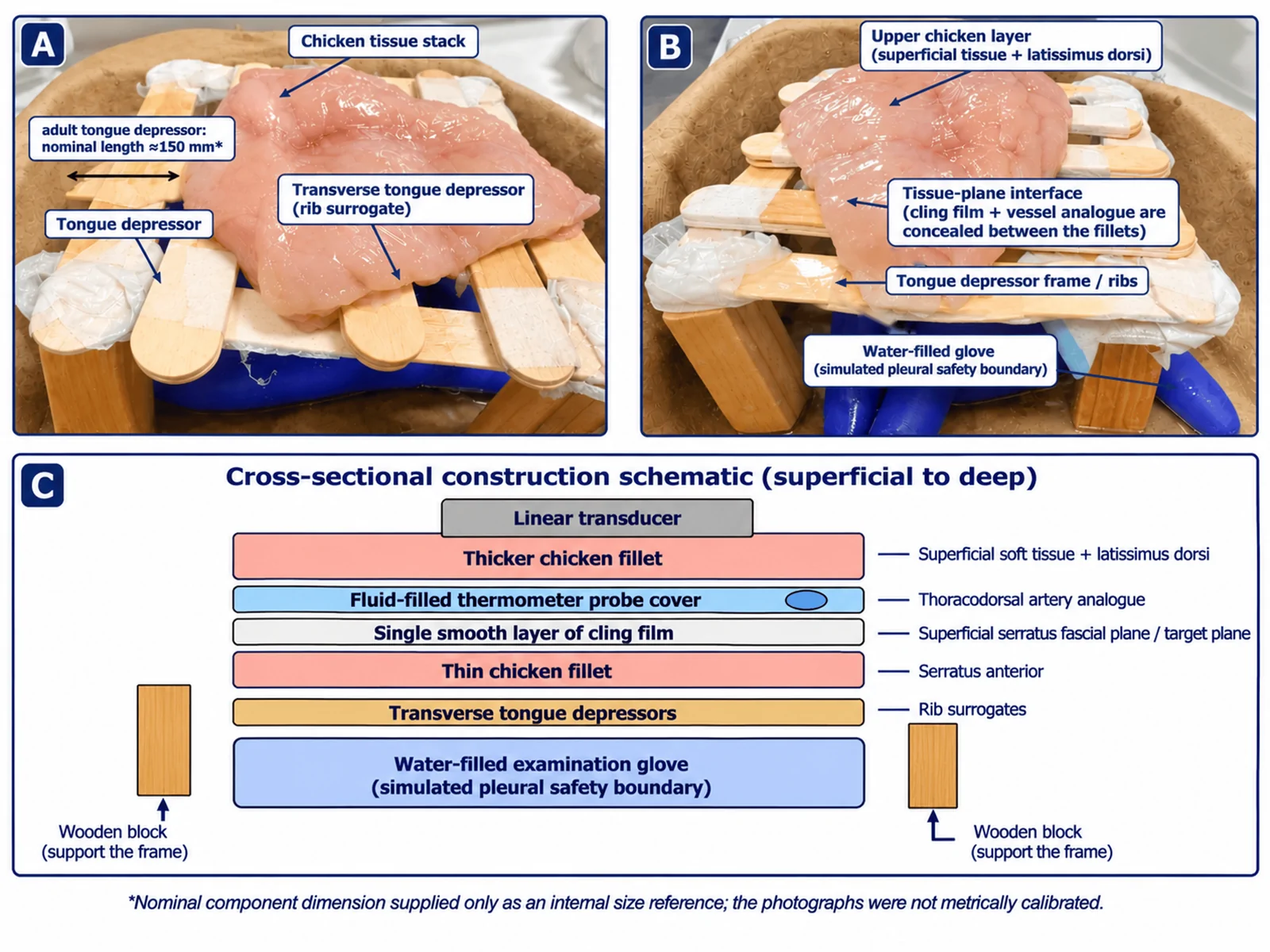
Construction of the low-fidelity serratus anterior plane block phantom Panel A shows the raised tongue-depressor scaffold supported by four wooden stacking blocks, with transverse tongue depressors acting as rib surrogates and a water-filled glove beneath. A standard adult tongue depressor (nominally approximately 150 mm long) provides an internal size reference; the photograph itself was not metrically calibrated. Panel B shows the completed tissue stack; the cling-film target plane and fluid-filled thermometer-probe-cover vessel analogue are concealed between the chicken layers. Panel C provides a labelled cross-sectional schematic of the construction. The water-filled glove is a simulated pleural safety boundary, not a representation of lung tissue.

**Table 1:** Bill of materials and proposed replication specification The values in Table 1 are intended to make future builds more standardised. They should not be interpreted as prospectively measured dimensions of the original proof-of-concept scaffold.

Component	Quantity/Material	Replication Specification
Support legs	Four identical rectangular wooden stacking-game blocks	Place vertically to create a rigid raised scaffold. Original block dimensions were not prospectively recorded; future builds should use four matched blocks approximately 70-80 mm high when vertical.
Frame	Standard adult wooden tongue depressors	Use approximately eight tongue depressors to form/reinforce a rectangular frame. A standard adult depressor is nominally about 150 x 18 mm; exact brand dimensions vary.
Rib surrogates	Three transverse tongue depressors	Place across the central opening at approximately 20-30 mm centre-to-centre spacing.
Pleural safety	One disposable examination glove filled with water	Fill until the upper glove surface is flush with the undersurface of the rib surrogates. Use contact rather than a fixed volume because glove and bowl geometry vary.
Serratus anterior	One thinner chicken-breast fillet	Trim while scanning so that, under light probe compression, the superficial target plane is approximately 8-12 mm deep and the rib level approximately 18-25 mm deep.
Fascial plane	One smooth single layer of cling film	Place between the two chicken layers. Eliminate folds and trapped air with a small amount of water, saline or ultrasound gel.
Thoracodorsal artery analogue	One fluid-filled thermometer probe cover segment	Use a segment long enough to cross the scanning window, approximately 60-100 mm, and secure it beyond the active scan field.
Latissimus dorsi/superficial tissue analogue	One thicker chicken-breast fillet	Place above the cling-film plane and vessel analogue.
Container and fixation	Waterproof vomit bowl or tray; adhesive tape	Keep raw poultry isolated from clinical work surfaces. The original assembly time was not prospectively recorded.

Four rectangular wooden stacking blocks were positioned vertically as legs. Standard adult wooden tongue depressors were taped across the blocks to form a rectangular platform. Three transverse tongue depressors were positioned across the central opening as rib surrogates, with a target spacing of approximately 20-30 mm between their centres for future builds. A water-filled examination glove was placed beneath the transverse tongue depressors and adjusted until its upper surface was in direct contact with their undersurface. The glove represented a simulated pleural safety boundary rather than lung tissue. 

The tissue stack was assembled from deep to superficial as follows: the water-filled glove; transverse tongue-depressor rib surrogates; a thinner chicken fillet representing serratus anterior; a single smooth layer of cling film representing the superficial serratus fascial plane; a water- or saline-filled thermometer probe cover representing the thoracodorsal artery; and a thicker chicken fillet representing latissimus dorsi and superficial soft tissues. A small quantity of water, saline or ultrasound gel was used between layers to minimise trapped air. The vessel analogue was secured beyond the active scanning window so that tape did not obscure the target plane. 

For future replication, rather than relying on raw fillet thickness, we recommend trimming the chicken while scanning until the superficial target plane appears at approximately 8-12 mm and the upper surface of the rib surrogates at approximately 18-25 mm under light probe compression. This approach uses the sonographic depth relationship as the reproducibility target and accommodates natural variability in chicken-breast thickness. The water-filled glove should be filled to achieve flush contact with the rib surrogates; a fixed volume is not recommended because glove size and bowl geometry vary.

At UK retail prices in August 2026, the consumable components (two chicken breast fillets, an examination glove, cling film, a thermometer probe cover and eleven tongue depressors) cost approximately £3.76 per model (Table 2), of which chicken accounted for £3.35; the wooden stacking-block scaffold and waterproof bowl are reusable and represent a one-off cost that was not itemised. Ultrasound equipment, needles, saline, adhesive tape and staff time are excluded.

**Table 2:** Itemised consumable cost per model Prices are UK retail prices from a supermarket (17 August 2026) and a medical supplier (AHP Medical). Reusable items (wooden stacking blocks, waterproof bowl), adhesive tape, ultrasound equipment, needles, saline and staff time are excluded.

Component	Quantity Used	Source and Pack Price	Cost per model (£)
Chicken breast fillets	2	Supermarket, £6.69/kg (4 fillets per pack)	3.35
Cling film	about 1m	Supermarket, 40m for £1.90	0.05
Examination glove	1	Supermarket, 40 for £5.95	0.15
Wooden tongue depressors	11	Medical Supplier, 100 for £1.36	0.15
Thermometer probe cover	1	Medical supplier, 250 for £15.94	0.06
Total consumable cost			3.76

Prototype characterisation 

The final prototype was scanned and needled by the senior author during one proof-of-concept bench session using a high-frequency linear transducer. Two archived still images were reviewed retrospectively against the on-screen depth scales. The superficial fascial target plane was approximately 8-10 mm deep, the vessel analogue occupied approximately the same 9-12 mm depth range, the upper surfaces of the tongue-depressor rib surrogates were approximately 18-22 mm deep, and the upper glove interface was approximately 23-28 mm deep. These are post hoc estimates from a single build, not repeated measurements. The target plane was, therefore, slightly shallower than, but broadly comparable with, the 10-20 mm depth described in the original human volunteer series [2]. 

The archived images demonstrate recognisable tissue-equivalent layers, needle visibility and depth relationships in this build (Figure 2). A small saline injection was used during proof-of-concept needling and qualitative separation of the simulated fascial interface was observed. However, the number of needle passes and injections was not prospectively counted, no predefined hydrodissection success criterion was used, and durability was not measured. The water-filled glove was incorporated so that excessive deep advancement could produce visible leakage, collapse or rupture, but the sensitivity and specificity of that feedback mechanism were not formally tested. No trainees were assessed, and no structured learner feedback was collected in this technical report. 

**Figure 2:**
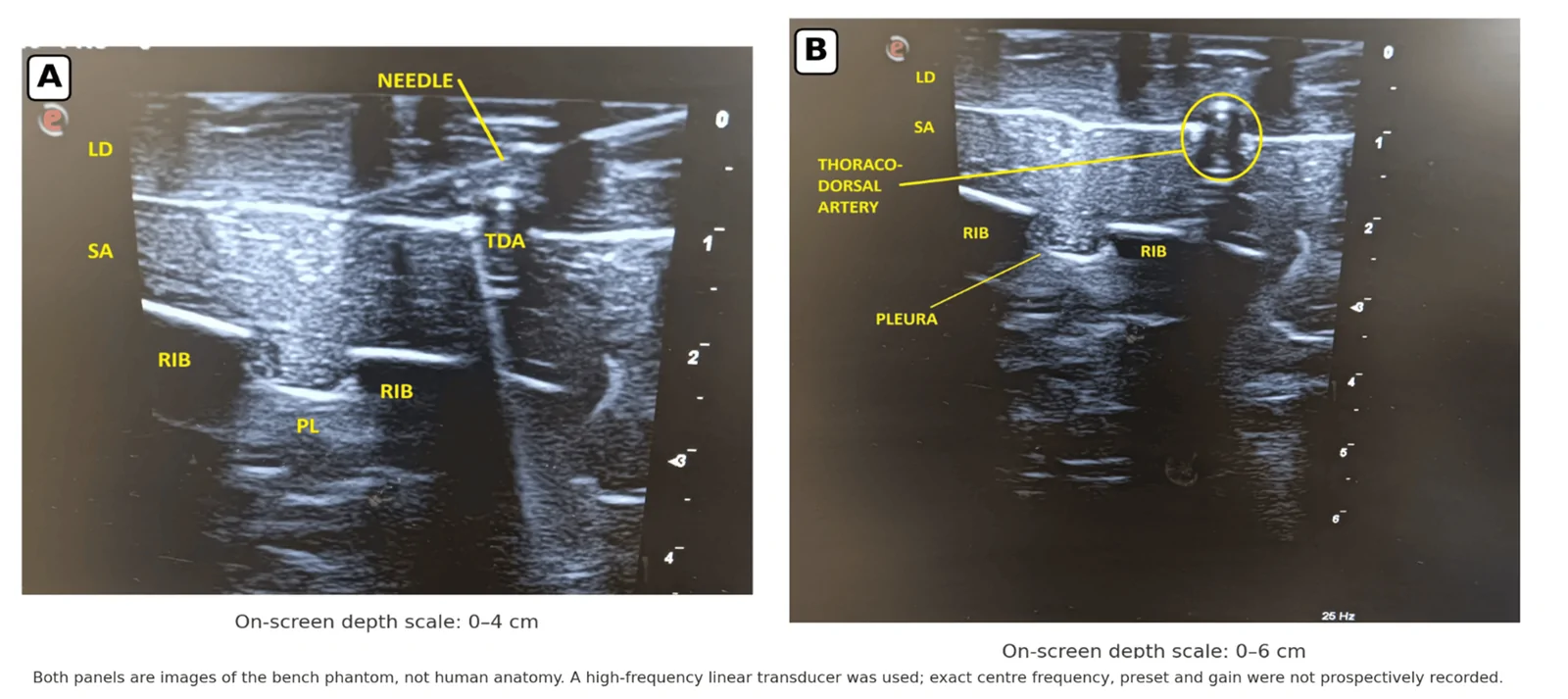
Ultrasound appearance of the author’s serratus anterior plane block phantom Neither panel shows human anatomy. Panel A shows in-plane needle advancement towards the superficial serratus plane. The on-screen depth scale extends to 4 cm. Panel B shows the same prototype at a 6-cm depth setting; the circled region is the vessel analogue, and the labelled RIB and PLEURA interfaces arise from the tongue-depressor/glove construct rather than human ribs or pleura. A high-frequency linear transducer was used. Exact centre frequency, preset and gain were not prospectively recorded and are therefore not reported. Abbreviations: LD, simulated latissimus dorsi; SA, simulated serratus anterior; TDA, fluid-filled thermometer-probe-cover thoracodorsal artery analogue; PL, simulated pleural safety boundary.

Accordingly, statements about model behaviour are deliberately framed as observed qualitative findings from one proof-of-concept session or as design intent. The model has not yet demonstrated reproducibility, educational effectiveness or transfer to patient care. 

Proposed training task and assessment 

The primary training task is a superficial SAPB. Learners first identify the two chicken layers, the intervening superficial serratus plane, the simulated ribs and the deep glove boundary. The fluid-filled thermometer probe cover represents the thoracodorsal artery and must be identified before needle insertion. On actual human scanning, this step should include colour Doppler confirmation of the thoracodorsal vessel. The phantom analogue is static and does not reproduce pulsatility or Doppler flow. 

The learner then plans an in-plane needle trajectory into the superficial fascial plane while maintaining continuous needle-tip visualisation. A small-volume saline test injection is used to assess whether the tip is within the intended interface. The design is intended to allow separation of the cling-film interface during hydrodissection. Contact with or penetration of the thermometer-cover analogue constitutes an unsafe vascular trajectory and requires the learner to stop and reposition. 

An optional deep-plane safety station may be added for learners who are also being trained in the deep SAPB approach. The needle is directed towards the plane between serratus anterior and the rib surrogates. The glove is positioned immediately beneath the tongue depressors so that advancement beyond the simulated rib level can puncture the glove and is designed to produce immediate visible or tactile feedback of a simulated pleural-boundary breach. This is a procedural safety cue only; the model does not reproduce pleural sliding, lung tissue, respiratory movement or pneumothorax physiology. 

Table 3 presents a proposed assessment framework. It uses three behavioural anchors: 0, not demonstrated or unsafe; 1, demonstrated only with prompting, correction or intermittent loss of control; and 2, demonstrated independently and safely. As an unvalidated operational proposal, knowledge and human sonoanatomy domains should be completed satisfactorily once, while the mandatory phantom domains should score 2 on two consecutive attempts before progression is considered. The deep safety station is optional when the local programme focuses on superficial SAPB and should not determine progression. Assessment should be performed by an appropriately credentialled local supervisor. These thresholds are proposed for standardisation and require prospective validation. 

**Table 3:** Proposed assessment domain before progression to directly supervised patient practice

Domain	Observable Criteria	Proposed Behavioural Anchor
Knowledge and governance	Explains indications, contraindications, consent, asepsis, monitoring, local anaesthetic dose limits, complications and local anaesthetic systemic toxicity management. States that simulation completion permits supervised practice only.	0 = not demonstrated/unsafe; 1 = prompting or correction required; 2 = independent and safe.
Human sonoanatomy	Identifies latissimus dorsi, serratus anterior, ribs and pleura on a human subject and demonstrates the thoracodorsal vessel using colour Doppler. No needle insertion.	0 = not demonstrated/unsafe; 1 = prompting or correction required; 2 = independent and safe.
Ergonomics and image optimisation	Aligns operator, patient/model, probe, needle and screen; selects an appropriate transducer, depth and gain.	0 = not demonstrated/unsafe; 1 = prompting or correction required; 2 = independent and safe.
Needle control	Uses an in-plane approach, maintains continuous needle-tip visualisation and stops/repositions if the tip is lost.	0 = not demonstrated/unsafe; 1 = prompting or correction required; 2 = independent and safe.
Vessel avoidance	Identifies the thermometer-cover vessel analogue and selects a trajectory that does not contact or puncture it.	0 = not demonstrated/unsafe; 1 = prompting or correction required; 2 = independent and safe.
Superficial-plane injection	Places the tip between simulated latissimus dorsi and serratus anterior and demonstrates appropriate plane separation after a small test injection.	0 = not demonstrated/unsafe; 1 = prompting or correction required; 2 = independent and safe.
Optional deep safety station	Identifies the simulated rib level and pleural boundary, avoids advancement beyond the rib surrogates and explains that glove puncture represents a simulated pleural-boundary breach, not pneumothorax physiology.	0 = not demonstrated/unsafe; 1 = prompting or correction required; 2 = independent and safe. Optional; does not determine progression in a superficial-SAPB programme.

The proposed use of Table 3 is as follows: an appropriately credentialed local supervisor should assess the learner. Knowledge and human sonoanatomy should be completed satisfactorily. For the mandatory phantom domains, a score of 2 on two consecutive attempts is proposed before progression to directly supervised patient practice. This threshold has not been validated and is offered only as an operational starting point. Final clinical entrustment requires patient-based workplace evidence and local sign-off. 

The proposed seven-step pathway is shown in Figure 3. It deliberately separates authentic human sonoanatomy recognition from psychomotor needling. Human scanning, without needle insertion, assesses recognition of latissimus dorsi, serratus anterior, ribs, pleura and the thoracodorsal vessel. Phantom assessment then evaluates probe-needle coordination, needle-tip control, vessel avoidance and fascial-plane injection. Successful preclinical performance permits progression only to directly supervised patient procedures. In a prospective evaluation, the same domains and anchors could be re-scored after a predefined interval without further practice to assess skill retention, and applied during direct observation of the first supervised patient procedures to assess transfer. Final entrustment and independent practice remain workplace decisions based on supervised clinical evidence and local governance. 

**Figure 3:**
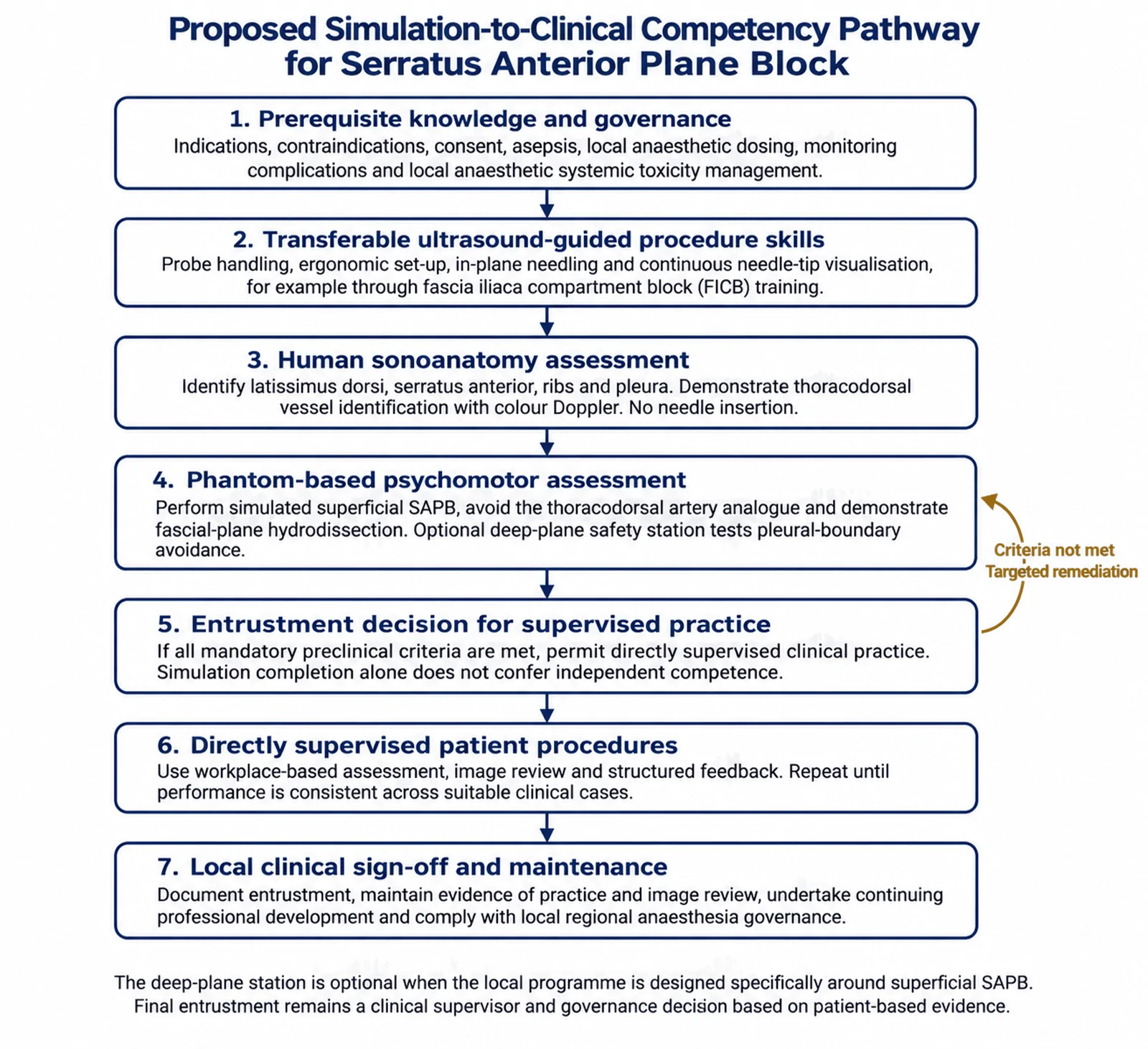
Proposed simulation-to-clinical competency pathway for ultrasound-guided serratus anterior plane block (SAPB). The pathway separates authentic human sonoanatomy recognition from phantom-based psychomotor assessment. The deep-plane safety station is optional when the programme is focused on superficial SAPB. If mandatory criteria are not met, targeted remediation returns the learner to phantom practice. Simulation is used as a gateway to directly supervised clinical practice and not as a substitute for patient-based entrustment.

## Discussion

This report describes a low-fidelity SAPB part-task trainer built from common materials and proposes how it could fit within a staged emergency medicine training pathway. Its intended contribution is not the use of chicken tissue itself, which is already well established [10-12], but the combination of a raised water-tolerant scaffold, synthetic rib surrogates, a named superficial vascular analogue and a deliberately vulnerable deep safety boundary. A ballistic-gel alternative may provide greater durability and standardisation where fabrication resources are available [13]. 

The thoracodorsal artery analogue is intended to make vascular avoidance an explicit task rather than an incidental discussion point. A learner could place the needle in the correct fascial plane yet still choose a trajectory through the vessel analogue. Requiring a safe approach, therefore, adds a decision-making component to the psychomotor task. However, because the analogue is static, it cannot replace human sonoanatomy scanning with colour Doppler. 

The pleural component should be interpreted with similar caution. The glove does not simulate a pneumothorax. It provides a deliberately vulnerable boundary immediately below the rib surrogates so that an unsafe deep trajectory can be made obvious to the learner. This emphasis is justified by the proximity and potential consequence of pleural injury, even though reported pneumothorax after SAPB is rare [5,6]. 

The staged pathway also reflects a broader principle in ultrasound-guided regional anaesthesia education: anatomy recognition and needle manipulation are related but separable skills. Simulation can improve knowledge and technical performance, although evidence for transfer to workplace performance and patient outcomes is less robust [14]. The proposed pathway, therefore, treats simulation as a gate to supervised clinical exposure, not as evidence of independent competence. 

Limitations 

This is a single-prototype descriptive technical report. The model has not undergone formal content, construct or predictive validity testing, inter-rater reliability assessment, durability testing or structured learner evaluation. Exact physical dimensions of the original wooden blocks, total tongue-depressor count in reinforcement layers, glove fill volume and assembly time were not prospectively recorded. Chicken thickness and tissue quality vary between builds, cling film may create an artificially bright interface, and repeated saline injection is expected to distort the plane. The archived sonographic depth measurements are post hoc estimates from two still images and do not constitute a formal depth study. The model does not reproduce skin puncture, respiratory motion, pleural sliding, vascular flow, patient movement or the physiological consequences of pneumothorax. The pleural-breach feedback mechanism has not been tested against a predefined number of deliberately safe and deliberately deep passes, so false-positive or false-negative leakage is unknown. No trainee progressed through the proposed pathway as part of this report. Future work should standardise the build dimensions and assembly time, quantify durability and breach feedback, and prospectively assess usability, inter-rater agreement, learner performance, skill retention and transfer to supervised clinical care. 

## Conclusions

The described phantom combines a chicken-based superficial SAPB target with a thoracodorsal artery analogue, synthetic rib surrogates and a water-filled deep safety boundary. In this single proof-of-concept build, archived ultrasound images showed a superficial target plane at approximately 8-10 mm and rib surrogates at approximately 18-22 mm. The model is designed to support rehearsal of in-plane needle visualisation, vessel avoidance and fascial-plane hydrodissection, with an optional deep station designed to make an unsafe pleural-boundary trajectory immediately apparent. 

The model, and the seven-step pathway proposed around it, are offered as a starting point for departments seeking to structure preclinical SAPB preparation. Whether the model improves learner performance, reliably reproduces pleural-breach feedback or can contribute to entrustment decisions has not yet been evaluated. Both the trainer and the proposed assessment thresholds require prospective validation before they can be relied upon to inform clinical sign-off.
